# Supplementary material for: Direct observation of photonic Landau levels and helical edge states in strained honeycomb lattices
Source: Light Sci Appl. 2020 Aug 19;9:144. doi: 10.1038/s41377-020-00377-6 (PMC7438334; doi:10.1038/s41377-020-00377-6)
Supplement: Supplementary file 1 — Supplemental Material [file 41377_2020_377_MOESM1_ESM.pdf]

# Supplementary material: Direct observation of photonic Landau levels and helical edge states in strained honeycomb lattices

Omar Jamadi,[1] Elena Rozas,[2] Grazia Salerno,[3] Marijana Milićević,[4] Tomoki Ozawa,[5] Isabelle Sagnes,[4] Aristide Lemaître,[4] Luc Le Gratiet,[4] Abdelmounaim Harouri,[4] Iacopo Carusotto,[6] Jacqueline Bloch,[4] and Alberto Amo[1]

[1]*Université de Lille, CNRS, UMR 8523 – PhLAM – Physique des Lasers Atomes et Molécules, F-59000 Lille, France*

[2]*Depto. de Física de Materiales e Instituto Nicolás Cabrera, Universidad Autónoma de Madrid (UAM), Madrid, 28049, Spain*

[3]*Center for Nonlinear Phenomena and Complex Systems, Université Libre de Bruxelles, CP 231, Campus Plaine, B-1050 Brussels, Belgium*

[4]*Université Paris-Saclay, CNRS, Centre de Nanosciences et de Nanotechnologies, 91120, Palaiseau, France*

[5]*Interdisciplinary Theoretical and Mathematical Sciences Program (iTHEMS), RIKEN, Wako, Saitama 351-0198, Japan*

[6]*INO-CNR BEC Center and Dipartimento di Fisica, Università di Trento, 38123 Povo, Italy*

(Dated: July 22, 2020)

# I. DESIGN OF THE UNIAXIAL STRAIN

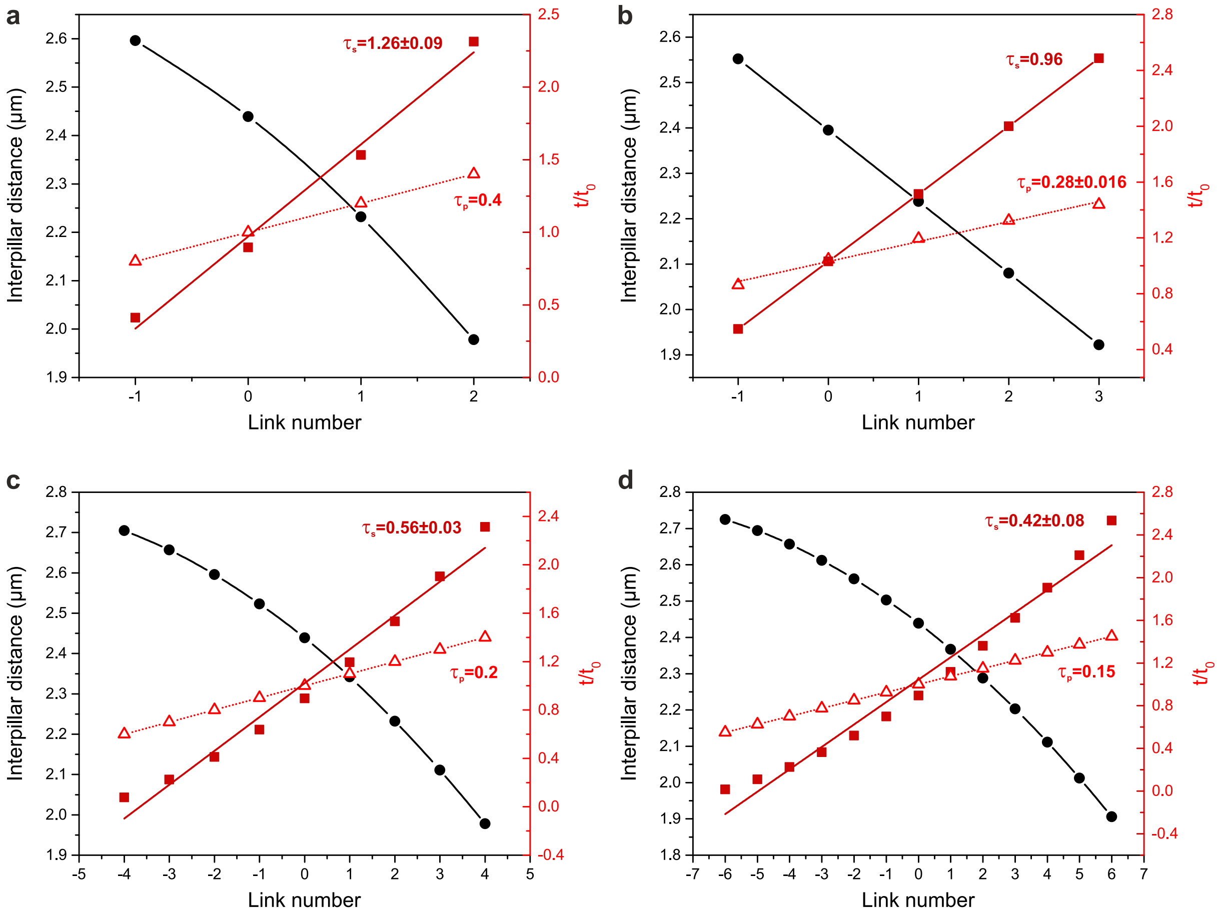


Fig. S 1. **Hopping gradient in the strained lattices employed in the experiments. a-d** Interpillar distance (black circles) and hopping variation $t/t_0$ along the strain gradient for $s$ (red squares) and $p$ bands (red empty triangles) as a function of the link number along the $x$ direction. Panel **a** refers to the lattice used in Figs. 2(e,f) and in Figs. 3(d,g) in the main text. Panel **b** refers to the lattice used in Fig. 1(c), Fig. 2(f), Fig. 3(d) and Fig. 4. Panel **c** refers to the lattice used in Figs. 2(b,d,f) and in Figs. 3(b,c,d,f,h). Panel **d** refers to the lattice used in Fig. 2(f) and Fig. 3(d).

## II. COLOURED VERSION OF THE TIGHT-BINDING BAND-STRUCTURE IN $S$ BANDS

Figure S2 shows a coloured version of the tight-binding band-structure used in Fig. 2(b) of the main text for the fit of the experimental data where the colour of each state is defined by the mean position of its wavefunction. Close to $E_0^s$ and the Dirac points $K$ and $K'$, the wavefunction of the Landau level $n = 0$ is localised in the bulk of the lattice (black region in Fig. S2). On the contrary, the bands extending away from the Dirac points correspond to states localised at the left and right zigzag edges.

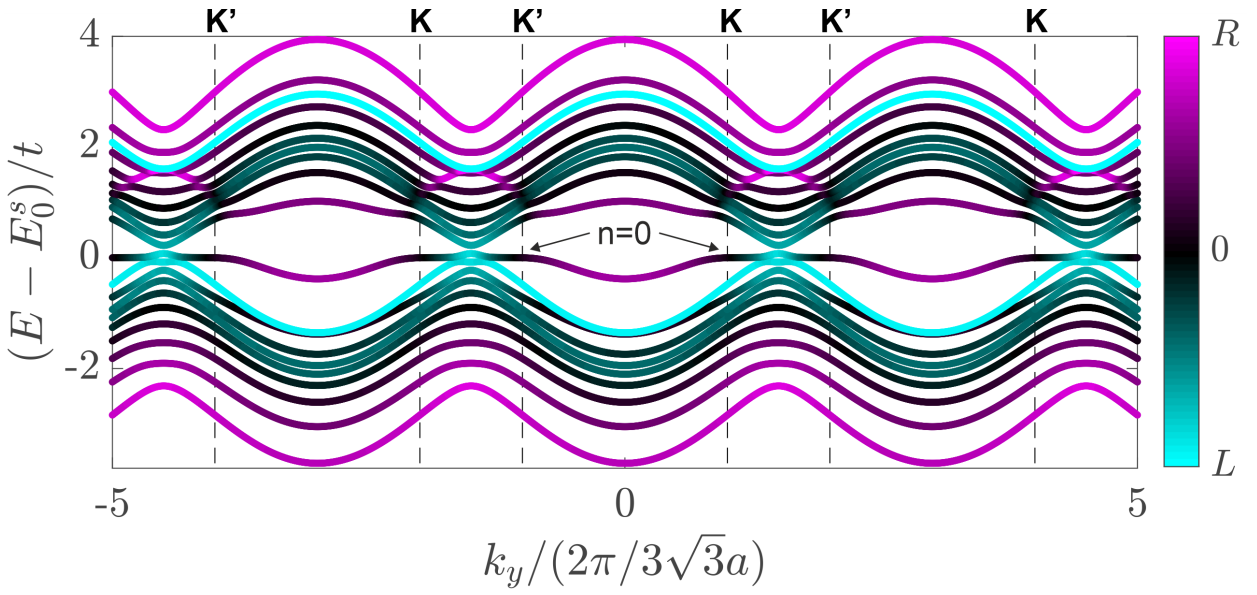


Fig. S 2. **Coloured version of the tight-binding band-structure in $s$ bands.** Tight-binding band-structure used in Fig. 2(b) of the main text for the fit of the spectrally resolved far-field emission for a strained lattice with a hopping gradient $\tau = 0.56$. The arrows indicate the position of the $n = 0$ Landau levels studied in the main text. The colours are defined by the mean position of the wavefunction of each state: magenta for states localised on the right edge, cyan for states localised on the left edge, and black for bulk states.

## III. COLOURED VERSION OF THE TIGHT-BINDING BAND-STRUCTURE IN *P* BANDS

Figure S3 shows a coloured version of the tight-binding band-structure used in Fig. 3(b) and in Fig. 3(c) of the main text for the fit of the $p$ bands where the colour of each state is defined by the mean position of its wavefunction.

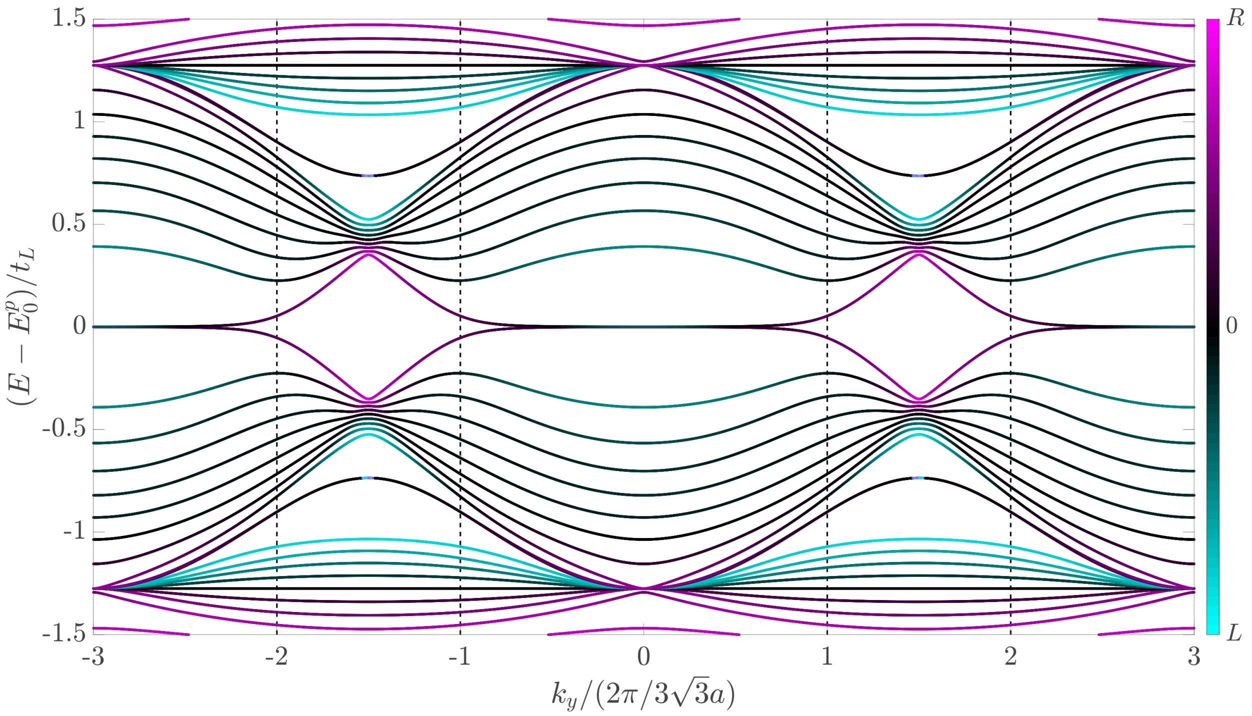


Fig. S 3. **Coloured version of the tight-binding band-structure in *p* bands.** Tight-binding band-structure used in Fig. 3(b) of the main text for the fit of the spectrally resolved far-field emission for a strained lattice with a hopping gradient $\tau = 0.2$. The colours are defined by the mean position of the wavefunction of each state: magenta for states localised on the right edge, cyan for states localised on the left edge, and black for bulk states.

## IV. MEASURED DISPERSIONS AND TIGHT BINDING BAND-STRUCTURES IN *S* BANDS (FOR $\tau = 1.26$) AND *P* BANDS ($\tau = 0.4$)

Figures S4(a) and (c) present the spectrally resolved far-field emission of the $p$ and $s$ bands for a lattice with a strain gradient $\tau = 0.4$ ($p$ bands) and $\tau = 1.26$ ($s$ bands). This

lattice corresponds to the one shown in Fig. 2(e) and Fig. 3(g) in the main text. The measured dispersions are qualitatively reproduced using the same procedure as in Fig. 2(b) and Fig. 3(b).

In $s$ bands (Fig. S4(c)), despite the strain being twice as large as in Fig. 2(b), the identification of the $n = 0$ Landau level still remains difficult due to complex finite size effects, the presence of an onsite energy offset on the edges and the broad linewidth of the emission ($\sim 200$ meV). In $p$ bands (Fig. S4(a)), the overlap between the edge states on opposite sides induces a splitting of the trivial zigzag edge states visible for $k_y \in [-2, -4]k_{y0} \cup [2, 4]k_{y0}$. Similarly to Fig. 3(b), two energies can be defined at the position of the Dirac points for the n=0 Landau level: $\epsilon^p_{0-} = E^p_0 - 0.1$ meV and $\epsilon^p_{0+} = E^p_0 + 0.1$ meV (see zoomed area in Fig. S4(b)). Compared to Fig. 3(b), the reduced size of the lattice results in a larger splitting of the $n = 0$ Landau level while the higher strain $\tau$ yields to a larger gap between the $n = 0$ and $n = -1$ Landau levels as shown in Fig. S4(b)). Finally, the helical propagating edge states are also visible close to the Dirac points in the gap between Landau levels $n = 0$ and $n = -1$, and between Landau levels $n = 0$ and $n = 1$.

## V. ENERGY SPECTRUM OF LANDAU LEVELS IN $P$ BANDS

To calculate the energy spectrum of Landau levels in the $p$ bands, we assume that the hopping between $p$ orbitals oriented perpendicularly to the link between adjacent micropillars to be zero[1]. Considering the hopping between orbitals oriented parallel to the links to be $t_1$ for horizontal links, and $t_2 = t_3 \equiv t$ for angled links, and a lattice spacing $a = 1$, the momentum-space Hamiltonian is:

$$H(\mathbf{k}) = \begin{pmatrix} 0 & 0 & t_1 + \frac{t}{2}e^{-i3k_x/2}\cos(\frac{\sqrt{3}}{2}k_y) & t\frac{\sqrt{3}i}{2}e^{-i3k_x/2}\sin(\frac{\sqrt{3}}{2}k_y) \\ 0 & 0 & t\frac{\sqrt{3}i}{2}e^{-i3k_x/2}\sin(\frac{\sqrt{3}}{2}k_y) & \frac{3t}{2}e^{-i3k_x/2}\cos(\frac{\sqrt{3}}{2}k_y) \\ t_1 + \frac{t}{2}e^{i3k_x/2}\cos(\frac{\sqrt{3}}{2}k_y) & -t\frac{\sqrt{3}i}{2}e^{i3k_x/2}\sin(\frac{\sqrt{3}}{2}k_y) & 0 & 0 \\ -t\frac{\sqrt{3}i}{2}e^{i3k_x/2}\sin(\frac{\sqrt{3}}{2}k_y) & \frac{3t}{2}e^{i3k_x/2}\cos(\frac{\sqrt{3}}{2}k_y) & 0 & 0 \end{pmatrix}. \tag{1}$$

The bare site energy is set to zero and the reduced Planck constant to unity ($\hbar = 1$). When

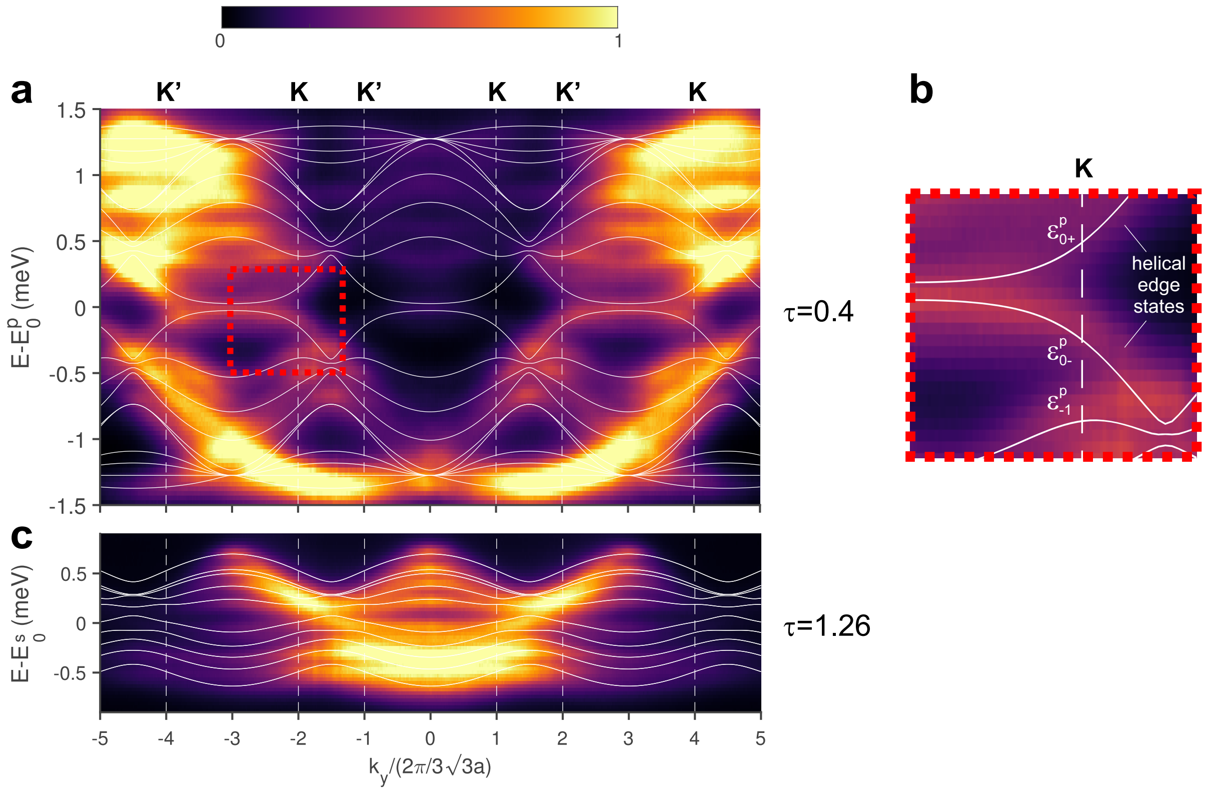


Fig. S 4. **Photonic Landau levels in $s$ and $p$ bands.** **a** Spectrally resolved far-field emission of the $p$ bands summed over all values of $k_x$ accessible in our setup, for a strained lattice with a gradient $\tau = 0.4$. The corresponding pseudomagnetic field is $B_z = 1000$ T. The Dirac energy is located at $E_0^p = 1570.75$ meV. **b** Zoom on the Landau levels $n = 0$ and $n = -1$. The white lines in **a**,**b** are theoretical fits using the tight-binding Hamiltonian with $t_L = -0.85$ meV and $t_T = 0$ meV. **c** Spectrally resolved far-field emission of the $s$ bands for a strained lattice with a gradient $\tau = 1.26$. The corresponding pseudomagnetic field is $B_z = 3200$ T. The Dirac energy is located at $E_0^s = 1567.9$ meV. The white solid lines are theoretical fits using the tight-binding Hamiltonian with $t = 0.17$ meV, $t' = 0$ meV, a hopping gradient $\tau = 1.26$, and an onsite energy of $+1.6t$ in the leftmost and rightmost pillar columns to account for the additional confinement of edge micropillars. The positions of the Dirac cones K and K' are indicated by vertical dashed lines.

$t_1 = t$, a Dirac point can be found at zero energy, for example, at $(k_x, k_y) = (0, -4\pi/3\sqrt{3}) \equiv$ $\mathbf{K}$. Expanding the Hamiltonian at this Dirac point and with $t = t_1$, we have :

$$H(\mathbf{K})_{t_1=t} = \frac{3}{4}t \begin{pmatrix} 0 & 0 & 1 & -i \\ 0 & 0 & -i & -1 \\ 1 & i & 0 & 0 \\ i & -1 & 0 & 0 \end{pmatrix}. \tag{2}$$

The eigenvalues of this Hamiltonian at the Dirac point are 0, 0, $-3t/2$, and $3t/2$. The corresponding normalized eigenvectors, in this order, are :

$$\frac{1}{\sqrt{2}}\begin{pmatrix} -i \\ 1 \\ 0 \\ 0 \end{pmatrix}, \qquad \frac{1}{\sqrt{2}}\begin{pmatrix} 0 \\ 0 \\ i \\ 1 \end{pmatrix}, \qquad \frac{1}{2}\begin{pmatrix} i \\ 1 \\ -i \\ 1 \end{pmatrix}, \qquad \frac{1}{2}\begin{pmatrix} -i \\ -1 \\ -i \\ 1 \end{pmatrix}. \tag{3}$$

In order to write the Hamiltonian in terms of the basis of these eigenvectors, we introduce a unitary matrix :

$$U \equiv \begin{pmatrix} \frac{-i}{\sqrt{2}} & 0 & \frac{i}{2} & \frac{-i}{2} \\ \frac{1}{\sqrt{2}} & 0 & \frac{1}{2} & \frac{-1}{2} \\ 0 & \frac{i}{\sqrt{2}} & \frac{-i}{2} & \frac{-i}{2} \\ 0 & \frac{1}{\sqrt{2}} & \frac{1}{2} & \frac{1}{2} \end{pmatrix}. \tag{4}$$

And then write the Hamiltonian in this basis as :

$$U^\dagger H(\mathbf{k}) U = \begin{pmatrix} \mathcal{H}_0 & \mathcal{C} \\ \mathcal{C}^\dagger & \mathcal{H}_e \end{pmatrix}. \tag{5}$$

Each block, $\mathcal{H}_0$, $\mathcal{C}$, and $\mathcal{H}_e$, is a 2-by-2 matrix. The effective 2-by-2 Hamiltonian $\mathcal{H}_0$ describes the physics around the zero energy Dirac point. The other block $\mathcal{H}_e$ describes what happens around the upper and lower energy regions $\sim 3t/2$ and $\sim -3t/2$. The block $\mathcal{C}$ describes the coupling between the zero energy region and the upper/lower energy region. To understand what happens around the Dirac point, we just need to focus on $\mathcal{H}_0$. Now letting $t_1$ be different from $t$, and expanding $\mathcal{H}_0$ around the Dirac point as $(k_x, k_y) = (0, -4\pi/3\sqrt{3}) + (q_x, q_y)$, one obtains, up to linear order in $(q_x, q_y)$ :

$$\mathcal{H}_0 \approx \frac{3}{4}t \begin{pmatrix} 0 & \left(-iq_x + q_y + \frac{2(t-t_1)}{3t}\right) \\ \left(iq_x + q_y + \frac{2(t-t_1)}{3t}\right) & 0 \end{pmatrix} = \frac{3}{4}t\left\{q_x\sigma_y + (q_y + 2(t-t_1)/3t)\sigma_x\right\}. \tag{6}$$

Writing this in a Dirac form $v_F\left[(q_x + eA_x)\sigma_y + (q_y + eA_y)\sigma_x)\right]$, we can read off that $v_F = 3t/4$ and :

$$eA_x = 0, \qquad eA_y = \frac{2(t-t_1)}{3t}. \tag{7}$$

If we assume that $t_1$ depends linearly on $x$ coordinate, as in the experiment :

$$t_1 = t\left(1 + \frac{x}{3}\tau\right), \tag{8}$$

one can find the expression of the pseudovector potential $eA_y = -2x\tau/9$ and the resulting pseudomagnetic field $eB = -2\tau/9$.

The off diagonal elements of (6) can be expressed as the following operator :

$$\hat{V} = -iq_x\frac{3t}{4} + q_y\frac{3t}{4} - \frac{x\tau t}{6}. \tag{9}$$

Using the canonical equation $[\hat{x}, \hat{q_x}] = i$, we have

$$[-\hat{V}, -\hat{V}^\dagger] = \frac{\tau t^2}{4}. \tag{10}$$

By defining $\hat{a} = -\hat{V}/(t\sqrt{\tau}/2)$, one can find the harmonic oscillator commutation relations $[\hat{a}, \hat{a}^\dagger] = 1$.

Setting $\omega \equiv t\sqrt{\tau}/2$, one can write

$$\hat{V} = -\omega\left(\sqrt{\frac{m\omega}{2}}(x - x_0) + i\sqrt{\frac{1}{2m\omega}}q_x\right) = -\omega\hat{a} \tag{11}$$

with

$$m = \frac{4\sqrt{\tau}}{9t}, \qquad x_0 = \frac{9q_y}{2\tau}. \tag{12}$$

The eigenvalue equation around the Dirac point can now be rewritten as

$$\mathcal{H}_0 \begin{pmatrix} \phi_A \\ \phi_B \end{pmatrix} = -\omega \begin{pmatrix} 0 & \hat{a} \\ \hat{a}^\dagger & 0 \end{pmatrix} \begin{pmatrix} \phi_A \\ \phi_B \end{pmatrix} = \mathcal{E} \begin{pmatrix} \phi_A \\ \phi_B \end{pmatrix}. \tag{13}$$

This eigenvalue equation can be solved in a way similar to the $s$ band Hamiltonian in Ref.[2]. The result is that the eigenvalues are relativistic Landau levels:

$$\mathcal{E}_n^p = \pm\omega\sqrt{|n|} = \pm\frac{t}{2}\sqrt{\tau|n|}, \tag{14}$$

where $n$ is any natural number.

## VI. SIMULATION OF PROPAGATION THROUGH HELICAL EDGE STATES

To confirm the presence of the $p$ bands helical edge states in our system, we performed driven-dissipative simulations of polariton lattices with the parameters of two strained lattices studied in our experiments.

We consider the strained lattices coherently driven by a monochromatic resonant pump with strength $F_p$ and frequency $\omega_p$. The dissipation is considered uniform for all lattice sites with a rate of $\gamma$. We search the steady states of the equation:

$$i\hbar\frac{\partial\psi}{\partial t} = (H_p - i\frac{\gamma}{2})\psi + F_p e^{i\omega_p t}, \tag{15}$$

where $H_p$ is the real space tight-binding Hamiltonian of the $p$ bands and $\psi$ is the vector containing the polariton amplitude on each site. To probe the propagating edge state of the zeroth Landau level, the pump is placed on one pillar near the zigzag edges and $\omega_p$ is set in between the $n = 0$ and $n = 1$ Landau level.

Figures S5(a) and (b) show the steady-state amplitudes for a system mimicking the strained lattice in Fig. 4(c,d) ($\tau = 0.28$, $B_z = 700$ T, $5 \times 18$ unit cells, 216 micropillars) with the pumped site, indicated by the arrow, respectively placed near the left and right zigzag edge. In full agreement with the experimental results shown in Fig. 4, the helical propagation of the $n = 0$ Landau level is only visible in the right zigzag edge.

If we consider the same system with $\tau = 0$, i.e. without any synthetic magnetic field $B_z$ (Figs. S5(c,d)), the emission from the left and right edge becomes symmetric, since now only trivial zigzag edges states mixed with bulk states are excited.

The same comparisons realized for a system mimicking the strained lattice in Figs. 3(f,h) ($\tau = 0.2$, $B_z = 500$ T, $10 \times 18$ unit cells, 360 micropillars) are shown in Figs. S6(a-d).


[1] M. Milićević, T. Ozawa, G. Montambaux, I. Carusotto, E. Galopin, A. Lemaître, L. Le Gratiet, I. Sagnes, J. Bloch, and A. Amo, "Orbital Edge States in a Photonic Honeycomb Lattice," Phys. Rev. Lett. **118**, 107403 (2017).

[2] Grazia Salerno, Tomoki Ozawa, Hannah M. Price, and Iacopo Carusotto, "How to directly observe Landau levels in driven-dissipative strained honeycomb lattices," 2D Mater. **2**, 34015 (2015).

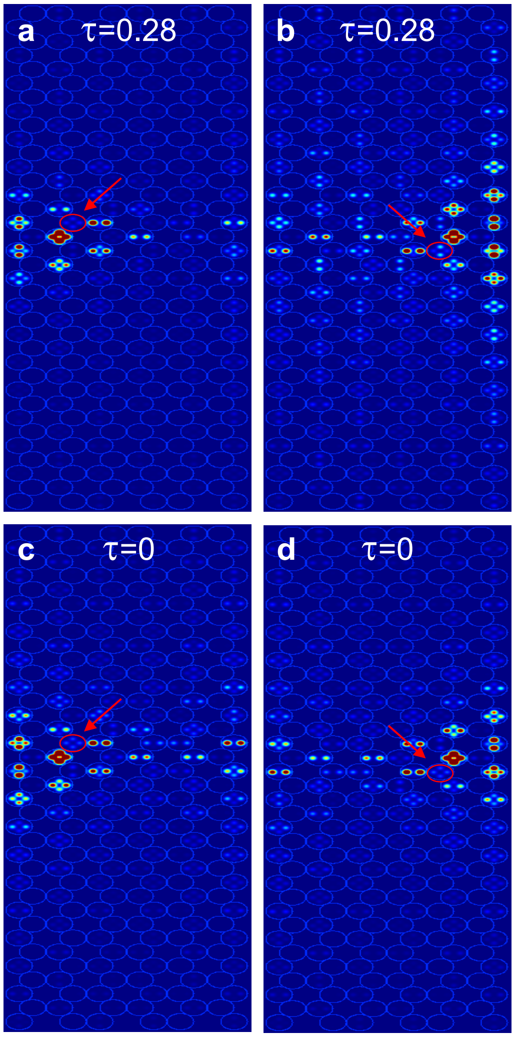


Fig. S 5. **Comparison of the steady state intensities for a strained ($\tau = 0.28$) and unstrained lattice. a,b** Polariton intensity distribution in the steady state under resonant pumping for a left **a** and right **b** edge excitation. The hopping gradient $\tau = 0.28$ considered in the calculations is the same as the one in lattice shown in Fig. 4 of the main text. The pump frequency is located in between the $n = 0$ and $n = 1$ Landau levels of the $p$-bands ($\omega_p = (\epsilon^p_{0+} + \epsilon^p_1)/4$) and the dissipation rate is equal to $\gamma = 0.2t$ ($t = 0.17$ meV). **c,d** Same calculations for $\tau = 0$. For each panel, the pumped site is indicated by an arrow and the strength of the pump is equal on $p_x$ and $p_y$ sub-states.

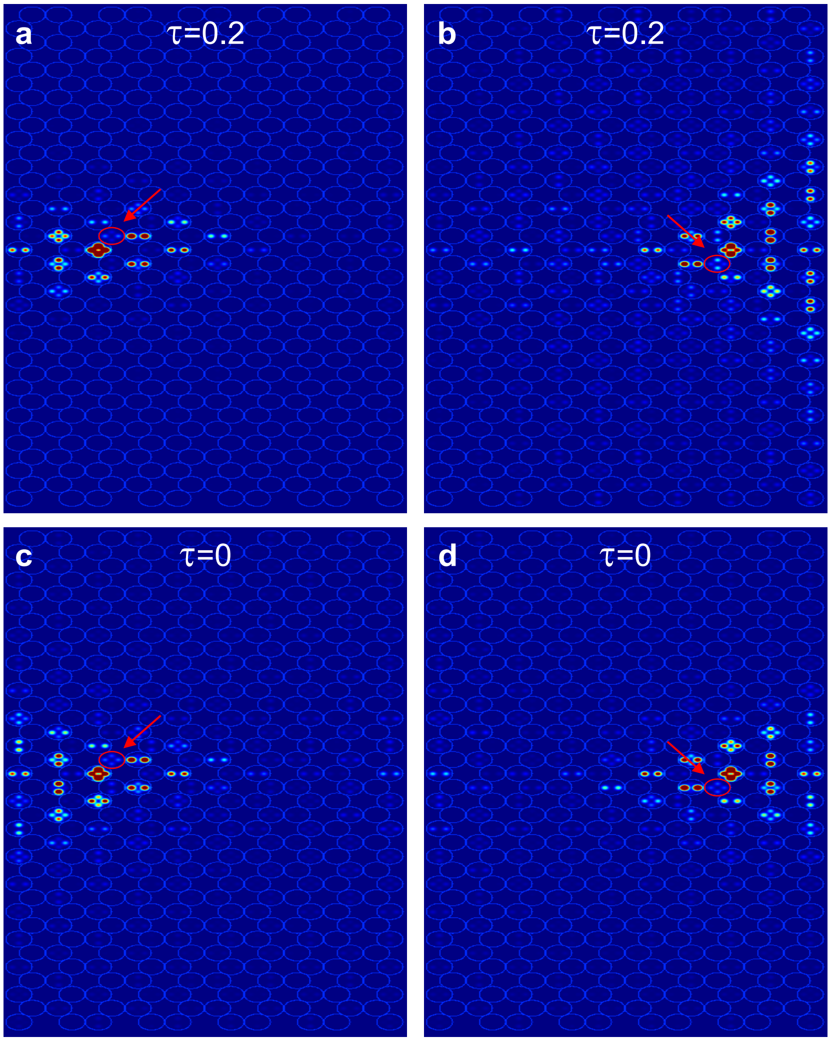


Fig. S 6. **Comparison of the steady state intensities for a strained ($\tau = 0.2$) and unstrained lattice. a,b** Polariton intensity distribution in the steady state under resonant pumping for a left **a** and right **b** edge excitation. The hopping gradient $\tau = 0.2$ considered in the calculations is the same as the one in lattice shown in Fig. 3(f,h) of the main text. The pump frequency is located in between the $n = 0$ and $n = 1$ Landau levels of the $p$-bands ($\omega_p = (\epsilon^p_{0+} + \epsilon^p_1)/3$) and the dissipation rate is equal to $\gamma = 0.2t$ ($t = 0.17$ meV). **c,d** Same calculations for $\tau = 0$. For each panel, the pumped site is indicated by an arrow. The strength of the pump is equal on $p_x$ and $p_y$ sub-states.
